# In the Spirit of Art. Transdisciplinary Education on Empathy as a Virtue for Master’s Students in Medicine

**DOI:** 10.5334/pme.1728

**Published:** 2025-09-25

**Authors:** Anne-Fleur van der Meer, Marleen van Beek, Anne Gudde, Marloes van Helvoort, Jelle van Gurp

**Affiliations:** 1 The Scientific Centre for the Quality of Healthcare (IQ Health), Ethics of Healthcare group, Radboud University Medical Centre, Nijmegen, The Netherlands; 2 Department of Psychiatry, Radboud University Medical Centre, Nijmegen, The Netherlands; 3 Pro Persona, Tiel, The Netherlands; 4 Radboud University Medical Centre, Nijmegen, The Netherlands; 5 Department of Psychiatry, Radboud University Medical Centre, Nijmegen, The Netherlands; 6 Scientific Centre for the Quality of Healthcare (IQ Health), Ethics of Healthcare group, Radboud University Medical Centre, Nijmegen, The Netherlands

## Abstract

**Background & Need for Innovation:**

The importance of physician empathy for the quality of care is widely acknowledged, but comprehensive attention to the formation of empathy is often lacking in medical curricula.

**Goal of Innovation:**

In response to this incongruity, the aim was to develop education that contributes to the formation of empathy. In line with virtue-ethical theories, empathy is viewed as a character trait (virtue), developed by practicing. This includes acquiring knowledge of empathy as a virtue and understanding its demands in morally salient (clinical) situations (virtue literacy).

**Steps Taken for Development and Implementation of Innovation:**

Four transdisciplinary teaching modules were developed for medical master’s students during their four-week psychiatry rotation at Radboud University Medical Center in Nijmegen. Within each module, students (a) engage in discussions on empathy as a multidimensional virtue, (b) participate in exercises involving visual arts, literature, writing, drama, or clinical cases, followed by structured reflection, and (c) apply the insights gained during their rotations and subsequently reflect on these experiences.

**Evaluation of Innovation:**

Learning outcomes were exploratively assessed through qualitative, thematic analyses of: 1) pre- and post-module written students’ accounts; 2) recordings of student discussions after modules; and 3) interviews on experiences and self-reported learning outcomes of the drama module.

**Critical Reflection on Process:**

The modules promote understanding of empathy as a multidimensional virtue and student reflection on their empathic attitudes in clinical practice. Key factors include using art, connecting strongly to clinical experiences, and co-teaching by an ethicist or medical humanities teacher and a psychiatrist. More guidance (e.g. homework) may further support learning.

## Background & Need for Innovation

The importance of physician empathy for the quality of care is widely acknowledged. Empathic physicians make more specific diagnoses, ensure that their patients are engaged in their treatment, and contribute to a strong therapeutic relationship [1,2]. Medical students are reminded of the essentiality of being empathic throughout their education.

Against this background, one would expect empathy to hold a prominent place in medical master programs. However, systematic and comprehensive attention to empathy is often lacking in medical curricula. The Radboud University Medical Center (Radboudumc, Nijmegen, the Netherlands) is, despite its strong emphasis on personalized care [3], no exception to this: empathy is mainly a side note during classes on communication skills or one of the topics in general ethics classes. The adverse consequence of this likely is that students feel they *should be* empathetic without knowing *how*, or that they experience what has been called ‘empathic dissonance’: the discomfort created by the act of making expressions of empathy that are not sincerely felt [4].

In our project, a collaboration between the Radboudumc ethics- and psychiatry departments, we developed four interconnected teaching modules (see Table 1) on empathy for master students in medicine who participate in a four-week psychiatry clerkship. The choice for psychiatry was deliberate: it is a field where it is of particular importance to be able to relate empathetically to others, including in difficult situations where mental health issues might interfere with patients’ ability to communicate about their feelings logically and emotionally [5].

**Table 1:** Overview of our interconnected three-hour teaching modules and learning goals.


MODULE	CONTENT AND METHODS	LEARNING GOALS *AFTER THE MODULE STUDENTS CAN:*	MATERIALS

1. Story	Introduction to module, relevance and learning goals Discussion on empathy as a virtue, led by teachers Analysis of poetry and fictional representations of experiences of people with mental health issues. Questions such as: ‘What is going on with(in) the main character, what do you (need to) do to find out?’ Individual and plenary reflections on experiences with trying to understand and empathize with characters and situations	describe four different dimensions of empathy (and its interconnectedness with other virtues), affective and cognitive dimensions in particular. can analyze and make sense of, on a basic level, the (unconventional) language and behaviors that express experiential and thought worlds of psychiatric patients (as depicted in literature and art). reflect on their experiences with trying to engage empathically with these worlds, affective and cognitive dimensions of empathy in particular. in line with 1–3, reflect on what different dimensions of empathy might ask of them in psychiatric practice, affective and cognitive dimensions in particular.	Insights on empathy from virtue-ethical literature discussed by teachers and integrated in Figure 1. Various types of literary texts and artworks (all representing themes relevant for psychiatry), mixing relatively accessible and coherent poetry (with some distinctive metaphors), with more incoherent/opaque texts. Song text/poem ‘A2’ (2014), Romeijn, printed on A3 Excerpt of novel *Weerlicht* (2022), Wortel. Excerpts of graphic novel *Lighter than my shadow*, (2013) Green.

2. Imagination	Recapitulation of module 1, students’ reflections on experiences with empathy during the rotation Analyses of visual art and fiction followed by creative writing exercise (interior monologue) in which students are invited to use own words for another one’s experiences. Plenary reflection led by the teachers who connect students’ experiences with the exercise to theoretical insights into empathy and to students’ clinical experiences.	Same learning goals, but with a special focus on being able to describe and reflect on what it means – and asks of them – to engage empathically with ‘experiences’ more *obscure* or even *highly unfamiliar* to them.	Insights on empathy from virtue-ethical literature discussed by teachers, and integrated in Figure 1 (Supplementary file 1). Painting ‘Residents of the Willem Arntsz House’ (1924), Toorop Exerpts from ‘The Murder of a Buttercup’ (1913), Döblin

3. Play	Entering an unfamiliar place, viewing a live theater performance based on personal experiences with psychiatry of the actress, exercises in sharing and listening to personal experiences.	describe four different dimensions of empathy (and its interconnectedness with other virtues), physical dimensions in particular. describe attitudes and language that may enable or hinder empathetic engagement reflect on their experiences with different dimensions of empathy (physical dimensions of empathy in particular) and on what this might ask of them in clinical practice.	A suitable and inspiring place for performing drama and telling stories, in our case: Theater Roest, Nijmegen.

4. Deliberation	Recapitulation of module 1, 2, 3; students’ reflections on experiences with empathy during the rotation Guided discussion of a case involving psychiatric experiences and a moral dilemma, exercises to comprehend and describe the experiences and dilemma thoroughly and make a deliberate decision. Plenary reflection led by the teachers who connect students’ experiences with the exercise to theoretical insights into empathy and to students’ clinical experiences	describe how empathy (and its interconnectedness with other virtues can be the starting point for (moral) decision making in psychiatric practice. describe ways of emotional involvement that may enable or hinder empathy and thus good (moral) decision making reflect on their experiences with different dimensions of empathy while making moral decisions, and on what this might ask of them in psychiatric practice.	Case description, following a particular timeline. Cards with different roles involved in the case (resident doctor, psychiatrist, psychiatric nurse, mayor, family member). Students will take these roles during the exercise.


Two aspects stand out about our modules, as we will elaborate later: they are transdisciplinary and they are founded on the concept of empathy as a multidimensional virtue. At its core is the understanding that being empathetic requires *more* than the competent performance of a communication skill. At the same time, it should also be *less* than the complete identification with a patient’s suffering. Educating for empathic doctors requires that students learn to develop personal qualities that enable them to take the right (e.g. balanced) caring attitude, that guide them to make considerate decisions and act accordingly [6]. This view is central to the approach of virtue ethics, an ethical approach that regards moral (*good*) behavior as arising from character traits cultivated throughout one’s life by an interaction of i.e. habituation, learning from role models and reflection, such as courage, justice, integrity, and empathy. These character traits are called virtues which assist (young) physicians in specific situations to do the right thing, at the right time, and for the right reasons [7], thereby enabling them to provide high-quality, personalized care [8].

## Goal of Innovation

The aim of our innovation was to develop comprehensive education that contributes to the formation of empathy. In line with virtue-ethical theories, we consider empathy a character trait (virtue) that can be cultivated throughout one’s life by practicing [8,9]. This practice includes acquiring knowledge of empathy as a virtue and understanding its demands (from character and personal qualities) in morally salient (clinical) situations [10,11]. In virtue-pedagogical literature this knowledge and understanding is referred to as ‘virtue literacy’ and considered a key prerequisite for virtue formation [10,11]. It is the formation of virtue literacy that our modules aim to stimulate.

**Figure 1:**
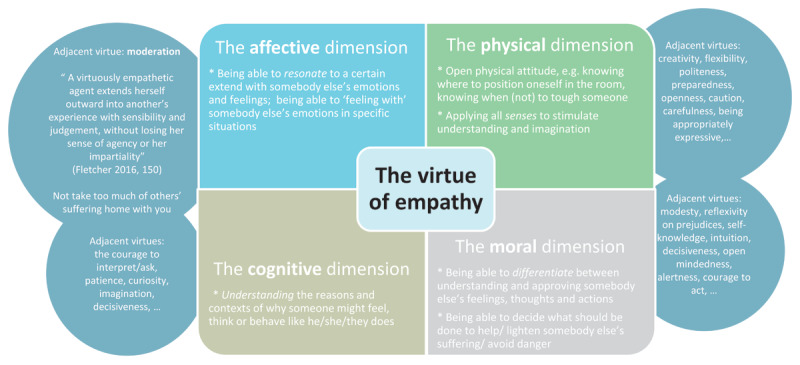
Empathy schematically conceptualized as a multidimensional virtue, as discussed in our four course modules.

The cultivation of virtue literacy has been described as unfolding in different stages [11]. An important first stage is the acquisition of knowledge of the virtue. In line with this, our first subgoal has been to enable students to become familiar with the virtue of empathy in its multiple facets. This means, that we aim to help students understand that empathy is a virtue with four dimensions: a cognitive (requiring the rational understanding of another’s situation), an affective (involving emotional resonance with the other), a physical (calling for appropriate bodily presence and positioning), and a moral dimension (demanding careful judgment about the appropriate course of action) [4,12,13,14,15]. In line with this, we teach that the virtue of empathy is connected with many other virtues (so called ‘adjacent virtues’): being virtuously empathic is impossible without being i.e. modest, open, curious, patient, decisive, and moderate [15]. See Figure 1 for a schematic overview of empathy as explored throughout our modules, drawing on our integration of theoretical perspectives that conceptualize empathy as a multidimensional virtue.

A second stage of developing virtue literacy entails encouraging students to apply virtue-related concepts to practical situations, typically demonstrated through writing or speaking about personal experiences [11]. In line with this, our second aim (subgoal 2) was to create opportunities for students to learn to reflect on how they enact empathy as a multidimensional virtue in the context of psychiatric practice, and to consider what this practice demands of them in morally salient situations – for instance, drawing upon other virtues as well. Here our goal is to realize the principles of experience-based learning, typically defined as a learning process that allows students to apply knowledge and insights in relevant real-world contexts [16].

## Steps taken for Development and Implementation of Innovation

### Context

The modules were conducted at the medical school of the Radboudumc in Nijmegen, Netherlands. The last three years of the 6-year medical program at the Radboudumc consist of clinical rotations in a fixed order, with groups of 25–30 students divided across several hospitals. The psychiatry clerkship is the third rotation and can be completed at different sites, including Radboudumc. A formal teaching program, both before and after the rotations, is mandatory for all students. During the 2022–2024 academic years, we designed and taught four interactive modules, each lasting three hours, for medical students during their psychiatry clerkship. The modules were a mandatory part of their master’s program (all students, regardless of motivation/affinity, attended the courses). The sessions took place weekly over a four-week period, allowing students to reflect in class on their clinical encounters and apply in practice what they learned during our course. With this, we aimed to achieve ‘just-in-time learning’, allowing students to apply the insights they have gained at the moment they are needed [17]. We conducted these series four times with groups of eight, six, six, and eight students respectively, mostly fourth year medical students, four of them were senior (sixth-year) medical students.

### Designing four interdisciplinary modules

The modules were designed to provide students with the opportunity to (1) acquire knowledge of virtue terms relevant to empathy and (2) apply, discuss, and reflect on them in real-life contexts. This was pursued by: (a) discussing theoretical insights on virtue ethics, focusing on empathy as a multidimensional virtue; (b) offering opportunities for students to practice empathy through exercises followed by reflection; and (c) enabling students to apply insights during clinical rotations and reflect on these experiences in subsequent sessions. These components were integrated into all four modules (see Table 1).

### Selection of practice materials

To support students in practicing empathic attitudes, we primarily used materials from literature and visual arts. Drawing on insights from the Medical Humanities and Art-Based Learning [18,19,20], we assumed these sources would stimulate students to explore empathy’s cognitive, affective, and moral dimensions, relevant to psychiatric practice; through close analysis of poems, novels and paintings students practice interpreting ambiguous and multilayered (visual) language, relating to conflicting perspectives and, based on that, considering what kind of personal and professional attitude might be required of them. Working with visual art and literature also means unraveling difficult words and metaphors often articulated by unconventional characters. It is for these characteristics that education in arts and literature is considered essential for future doctors, as the qualities they develop through such education prove invaluable in the clinical setting [18,20].

### Implementation- and teaching team

A transdisciplinary team of psychiatrists (in training), ethicists, and a literary scholar was assembled, often joined by a student in medicine for development and implementation. Each teaching session was led by experts in medical humanities and psychiatry. This collaboration was facilitated by established connections between the ethics and psychiatry departments. Due to a lack of in-house expertise, we engaged an external theatre company.

## Outcomes of evaluation

We exploratively assessed the outcomes of our modules by qualitative analyses of:

1. Four pre-and post-module written student accounts around each psychiatry rotation (concerning empathy-questions related to short excerpts from documentaries depicting individuals with mental illnesses). With help of ATLAS.ti, we compared course participants’ (based in the Radboudumc) answers to responses of students who did not participate in our course (e.g. Radboudumc-students who did their psychiatry clerkship elsewhere during the same period).
2. Recordings of our discussions with students on their experiences and reflections on the learning goals after each module interviews with students on their experiences and
3. Self-reported learning outcomes of module 3.

This allowed us to formulate tentative ideas about the learning outcomes of our modules, outlined in Table 2. In summary, students distinguish more explicitly between different dimensions of empathy as a virtue. They are most clear about the affective, cognitive and moral dimensions; mentioning the physical dimension is still limited. They have become aware of the dynamic aspect of empathy; the courage it requires to take an active interpretive step and the value of using creativity and imagination. They seem to have learned that in doing so, they need to strike a precarious balance (the virtue of moderation) between relying on intuition on the one hand and avoiding prejudice on the other.

**Table 2:** Exploratory overview of tentative learning outcomes from the modules, with relevant student quotes.


OUTCOMES	STUDENT QUOTES

**Goal 1: students can gain knowledge about empathy as a multidimensional virtue**	

**Students frequently mention realizing that education on empathy is limited in the curriculum**	“I don’t think we normally consider how much there is to say about empathy. If you had asked me before the clerkship… ‘What are we going to talk about for twelve hours?’ But really, we have no idea, and it doesn’t come up in the bachelor’s program either.” (interview after Module 3)

**Students report or show a richer understanding of empathy, often expanding their definitions to acknowledge it as a multidimensional character trait/virtue. In contrast, the students who did not participate in the module made no changes to their understanding**.	“Empathy consists of multiple aspects, including a cognitive and an affective component.” (from student response post module) “The cognitive and affective are two dimensions of empathy, and when something is very far removed from you, you can still try to understand it cognitively without necessarily feeling it emotionally.” (discussion after Module 2) “My understanding has become broader. […] [N]ow we’ve also learned about elements of feeling along with someone, and that it is a character trait that can grow over time.” (discussion after Module 3)

**Our teaching helped students recognize *adjacent* virtues related to empathy, such as imagination and the ability to defer judgment**.	“I have learned to look/listen to someone’s story with a more open mind and more curiosity.” (from student response post module) “Being empathetic—without judgment—is easier when you really know someone’s story.” (discussion after Module 4) “We learned that you have to strike a balance, not go too far or do too little.” (interview after Module 3)

**Goal 2: Students are enabled to reflect on their own empathic attitude in real life contexts**	

**Awareness of situations in which they (need to) use empathy during rotation**	“At the start of my clerkship, I was unconsciously using empathy, but I became much more aware of it—for instance, by setting aside my own prejudices in time and listening to the patient with curiosity.” (student response post module)

**Students discover that empathy is not static, but dynamic—something that can be present to varying degrees and can develop with practice**	“I sometimes find it difficult to understand psychiatric patients, for example someone with delusions. But it always evokes something in you, and from that point you can try – perhaps indirectly – to put yourself in the patient’s shoes and imagine something of it. I had the same with that first poem. At first I thought: what is going on here? But when you delve deeper and reflect on it, you can still draw meaning from it.” (discussion after Module 1)

**Awareness of the role of language and visual elements in empathy**	“By practicing empathy with both texts and images, I’ve learned that I need visuals to be optimally empathetic. I want to see a face, expressions. Language helps, but it’s not enough for me to get the full picture. That’s what I learned about myself.” (discussion after Module 4) “I need language to confirm what I see. An image alone isn’t enough to know.” (discussion after Module 2) “Every word and every word combination has meaning, so it really matters how a patient conveys something to you and how you convey something to a patient.” (discussion after Module 1)

**Awareness that people can experience, interpret, and evaluate the same situations differently**. **Note:** Several students indicated that this insight taught them a key condition for being empathetic: the ability to step away from their own evaluation of a situation. Students frequently mentioned the exercises involving the poem (Romeijn), the novel excerpt (Doblin), and the painting (Toorop) as triggers for this realization.	“It was enlightening to see how, as a group of five, we could all do the same assignment, yet with very different interpretations, highlighting that everyone experiences the world uniquely.” (discussion after Module 1) “Working with the poem made me realize that we all see things slightly differently and that a single thing can be interpreted in multiple ways.” (discussion after Module 1) “Staying too close to our own perspective and making overly quick assumptions can hinder our ability to truly understand someone.” (discussion after Module 2) “I’ve become more aware that I must try to assume less and dare to ask questions instead to check if my assumptions are correct.” (discussion after Module 2)

**Students discover that empathy is also about finding a balance between trusting in their intuition about someone and avoiding prejudice**	“[I started asking myself:] how clearly can I really perceive someone if I don’t know what is mine and what belongs to the other? Was I closed off to the artwork because I don’t like this type of art? Or was the artwork itself inaccessible to me?” (discussion after Module 2) “I’ve learned that I need to be careful with prejudices. They can help me, but also cause harm. In the case study, for example, it could lead to initiating compulsory care too soon, before really understanding the patient’s story. You need to strike a balance.” (discussion after Module 4)

**Awareness of the importance of imagination and interpretation (having the courage to take an active interpretive step)**. **Note:** Throughout the course, we often discussed the tension – among other things, explored through the lens of the virtues of moderation and imagination – between holding back interpretation and daring to interpret, a dynamic clearly reflected in the student quotes	“Expressing empathy by attempting to articulate someone’s possible thoughts (usually invisible) is a way to make abstract suffering more tangible. This process requires the courage to imaginatively fill in thoughts.” (Student after completing the inner monologue exercise, Module 2) “No. I don’t think her eating disorder alone explains her behavior. I believe Emma thinks and feels the way she does because perhaps a lot happened in her childhood that led to a troubled relationship with food, something she now wants to avoid. Maybe she experienced traumatic events as a child, or perhaps her parents raised her in a certain way that slowly contributed to this.” (*Student expanding on their pre-test answer to the question ‘What’s going on with Emma?’—showing a dare to interpret*)


## Critical Reflection on our Process

### Relating theory and experience

Students report a richer understanding of empathy, often (re)defining it (in the post-module written accounts) as a multidimensional character trait. They furthermore reflect on their own empathic attitude, sometimes in clear virtue- related terms. In this sense, it can be argued that our modules may have contributed to our students’ virtue literacy concerning empathy, as intended. We regard this as the result of our efforts to continuously connect students’ learning-experiences – whether during engaging with art and literature or discussing their clinical clerkships – to virtue-ethical concepts. For instance, when a student struggled with a literary text and attempted to interpret its meaning, we reframed this experience as a challenge of the *courage* that is needed to use creativity and imagination, moral restraint, and decisiveness. This approach was even further intensified after we found, during the first iteration of the module, that students rarely make such connections on their own.

What we also learn from the outcomes and our own reflections, is that students primarily refer to the cognitive, affective, and moral dimensions of empathy (see Table 2), while the physical dimension remains largely unmentioned. As we interpret a four-dimensional idea of empathy as a sign of virtue literacy, our intended outcome has, in this respect, not been fully achieved. Reflecting on potential reasons for this, we considered the possibility that, as instructors, we provided insufficient theoretical input and virtue-related language specifically addressing the physical dimension. This was partly due to the specific design of Module 3, in which the focus was primarily on practical exercises and looking at a theatre play, with limited theoretical reflection afterwards. It is however, precisely the integration of theory and practice that seems to foster student insights on empathy, as suggested by the frequent reference to the multiple other dimensions of empathy in their responses (see Table 2). Upon reflection, we conclude that Module 3 would have benefited from a stronger theoretical embedding.

### Connection with students’ experiences in clinical practice

Each session began with a recap to help students connect insights on empathy to their recent rotation experiences, though many struggled to do so. Senior medical interns, with more clinical experience, were better at making these connections. As *all* students could benefit from making these connections – for it would enable them to apply their literacy in real-world contexts, i.e. learning ‘just in time’ [18,19] – we recommend providing more guidance. In evaluations, students suggested that concrete homework exercises encouraging them to analyze rotation experiences through the lens of what they have learning on empathy in class could be helpful.

### Working with art and literature

For most students, working with this material was initially unfamiliar and sometimes uncomfortable. Especially in Module 1, this often meant that we, as teachers, had to provide significant guidance and encouragement to help students initiate the analysis and discussion of the art works. This experience encouraged us to thoroughly familiarize ourselves with the works in advance, and to carefully identify the elements likely to stimulate discussion.

What we consistently observed was that students gradually became more skilled with and increasingly enthusiastic about working with art and literature. It proved helpful to begin with relatively accessible texts and to gradually introduce greater complexity. The selected texts effectively stimulated reflection on what it means – and what it requires of character – to gain understanding and affection for someone or something. The ambiguity and openness of the selected works to multiple interpretations played a key role in this (see also Table 2).

It is advisable to explicitly incorporate students’ experiences of difficulty or discomfort with literature and art into their learning process, recognizing these experiences as relevant when engaging with empathy. For instance, we connected it to the challenges that psychiatrists may encounter when initially trying to reach or ‘read’ individuals in the consultation room. In this way, the ‘difficulty in interpreting poetry,’ for instance, became not an obstacle, but an entry point for reflecting on what such situations ask of someone – personally and professionally.

### Collaboration and implementation

We greatly benefited from our diverse expertise, as clinical, ethical, and medical humanities knowledge complemented each other. We recommend that others who wish to work with these methods involve both a teacher from the field of ethics or medical humanities and a clinician. The former contributes to practical and theoretical knowledge of (virtue) ethics and educational methods on literature and the arts, while the latter is able to offer insight into the moral and linguistic complexities of clinical practice.

After four iterations, we are working to structurally integrate the modules into educational sessions before, during, and after the psychiatry clerkship. A key challenge is the relatively high staffing cost, ideally involving interdisciplinary teaching. Since Radboudumc-ethics education during the psychiatry rotation is already co-taught by a psychiatrist and an ethicist, this seems feasible. However, the drama module could not yet be permanently included, as it depended on external partners and funding. To ensure sustainability, long-term feasibility should be considered from the start.

Finally, our outcomes and teaching experiences show that students not only develop empathy-related literacy but also gain insight into related virtues. These are relevant beyond psychiatry and can benefit learners and teachers in other educational contexts. We have, for example, used methods from Modules 1 and 2 in empathy workshops for medical specialists, educators, and mentors. While we often reuse the same materials, other texts or artworks may be equally suitable, as long as they invite students to engage in interpretive thinking and grapple with the experiences of central characters.
